# Supplementary material for: Whole Cow’s Milk but Not Lactose Can Induce Symptoms in Patients with Self-Reported Milk Intolerance: Evidence of Cow’s Milk Sensitivity in Adults
Source: Nutrients. 2021 Oct 27;13(11):3833. doi: 10.3390/nu13113833 (PMC8621065; doi:10.3390/nu13113833)
Supplement: Supplementary file 1 [file nutrients-13-03833-s001.zip › nutrients-1427360-supplementary/Carroccio et al - Supplementary File 1 October 1 2021.pdf]

Hydrogen Breath Test for Lactose (LHBT)

LHBT was performed after an overnight fast of 10 to 12 hours; a standard dose was administered of 25 g of lactose suspended in 250 to 300 ml of water (0.30 mol per liter), as previously described (1). A baseline alveolar breath sample was taken before ingestion of the lactose solution. Breath samples were collected at 30-minute intervals over a period of three hours from the time of ingestion and the hydrogen concentration in each breath sample was measured using the 12 Quintron Microlyzer (Quintron Instruments, Milwaukee, WI). Subjects were instructed not to eat or drink anything until completion of the test.

Subjects with an increase in breath hydrogen concentration > 20 ppm over baseline concentration were termed “maldigesters”, and those with one or more symptoms were classified as “intolerant”. Subjects whose hydrogen concentration increased < 20 ppm over baseline were termed “digesters”, and those without symptoms were classified as “tolerant”. All the subjects undergoing the test were contacted after 24 hours to evaluate the possible appearance of the following symptoms after the lactose challenge: abdominal pain or cramps, bloating, flatulence and diarrhea (defined as an urgent, watery defecation) (2).

In subjects with a hydrogen breath test showing a normal lactose absorption capacity (alveolar hydrogen concentration < 20 ppm), we evaluated the ability of the colonic flora to produce hydrogen through fermentation in response to carbohydrate malabsorption. These subjects were recalled over the following 8 days and, following the same procedure as the lactose load, 12 g of lactulose suspended in 120 ml of water was administered and breath hydrogen excretion was measured. Lactulose is a non-absorbable disaccharide that is fermented by the same enzymatic pathway as lactose. Patients with a negative lactulose hydrogen breath test were excluded from the study as considered to be lacking in the colonic flora which ferments carbohydrates and produces hydrogen.

Other assays and definitions

Allergology tests. Serum specific IgE assays for casein, beta-lactoglobulin and lactalbumin, and/or skin prick tests to whole cow’s milk or milk proteins were performed in all patients, as previously described (3).

Immunological assays and HLA typing for Celiac Disease. All patients underwent serum anti-transglutaminase IgA and anti-deamidated gliadin peptide IgG assays performed using commercial kits and reference values as described previously (4).

HLA-DQ phenotypes were determined by PCR using sequence-specific primers, with a DR and DQ sequence-specific primer kit (Unipath SpA, Milan, Italy) or a rapid method (DQ-CD Typing Plus by BioDiaGene, Palermo, Italy).

Duodenal histology. Most of the patients who reported a concomitant wheat sensitivity and tested positive for DQ2 or DQ8 HLA haplotypes underwent endoscopy in accordance with our standard diagnostic protocol, and four to six biopsy specimens were obtained from the second duodenal portion. The slides were stained with hematoxylin and eosin and graded according to the standardized scheme reported by Oberhuber et al. (5). The number of intra-epithelial lymphocytes (IELs) per 100 villous epithelial cells (enterocytes, EC) was assessed by immunohistochemical staining: $CD3^+$ IELs were stained with Leu-4 monoclonal antibody. The upper limit of the reference interval in our laboratory was 25 IELs/100 EC: cases with <25 IELs/100 EC and villi/crypts ratio $\geq$2.5 were classified as Marsh 0, while those with $\geq$ 25 IELs/100 EC were classified as Marsh 1 (i.e., intra-epithelial lymphocytosis). Slides with a villus/crypt ratio <2.5 were classified as "villous atrophy". Furthermore, to exclude a CD diagnosis, in this group of patients, we performed the anti-endomysium assay in the culture medium of the duodenal biopsies; the patients who tested positives were considered "coeliacs", even if the villus/crypt ratio in the duodenal mucosa was normal. Consequently, these patients were not included in the SRMI group.

Other investigations. In all cases, the presence of concomitant organic gastrointestinal diseases was accurately excluded. Consequently, the diagnostic workup of the patients may have also included abdominal ultrasonography, colonoscopy, histological examination of the colonic mucosa, as well as a small intestine barium examination.

Definitions. Functional gastrointestinal diseases were diagnosed according to current standard criteria (6). Anemia was defined as hemoglobin levels < 12 g/dl in females and < 13 g/dl in males. Weight loss was arbitrarily defined as weight loss > 5% in the previous three months.

DBPC whole cow's milk challenge

It was performed with whole cow's milk or rice milk as the placebo. In brief, after overnight fasting, the patients received 200 ml of commercial whole cow's milk or a rice milk; to mask the taste, a cup of coffee (30 ml of "espresso") was mixed with both the milk types; experiments with ten healthy volunteers had shown that the subjects were unable to distinguish between the two types (data not shown). Both patients and researchers were blinded to the kind of milk administered, as they were prepared by a nurse not involved in the study and the sequence of administration of the milk types

was randomly determined by envelopes. Cow's milk or rice milk were given in the presence of a nurse and the patients remained under observation for three hours, after which they returned home. The second challenge with cow's or rice milk was performed one week after the first one and in all cases when the patients were asymptomatic and on a cow's milk-free diet.

During the 24 hours following the challenge, the patients were asked to record the onset and severity of any eventual symptoms: the patients completed a 100mm visual analog scale, with 0 representing no symptoms, which assessed overall symptoms and the specific symptoms they each reported. In details, they reported a score for the following: bloating, abdominal pain, urgency of evacuation, stool frequency; furthermore, median and range number of evacuations were recorded. We recorded also the onset of eventual extra-intestinal symptoms on challenges.

The challenges were considered positive when clinical reactions occurred (increase in VAS score >30).

REFERENCES

1) Carroccio A, Montalto G, Cavera G, et al. Lactose intolerance and self-reported milk intolerance: relationship with lactose maldigestion and nutrient intake. Lactase Deficiency Study Group. J Am Coll Nutr 1998; 17: 631-6.

2) Martini MC, Savaiano DA. Reduced intolerance symptoms from lactose consumed during a meal. Am J Clin Nutr 1988; 47: 57-60.

3) Iacono G, Cavataio F, Montalto G, et al. Intolerance of cow's milk and chronic constipation in children. N Engl J Med 1998; 339: 1100-4.

4) Carroccio A, Mansueto P, Iacono G, et al. Non-Celiac wheat sensitivity diagnosed by DBPC challenge: exploring a new clinical entity. Am J Gastroenterol 2012; 107: 1898-906.

5) Oberhuber G, Granditsch G, Vogelsang H. The histopathology of coeliac disease: time for a standardized report scheme for pathologists. Eur J Gastroenterol Hepatol 1999; 11: 1185-94.

6) Drossman DA, Chang L, Chey WD, et al. The Rome IV Committees. Rome IV functional gastrointestinal disorders: disorders of gut-brain interaction. I. Raleigh, NC: The Rome Foundation; 2016.
