# Supplementary figures and images for: Whole Cow’s Milk but Not Lactose Can Induce Symptoms in Patients with Self-Reported Milk Intolerance: Evidence of Cow’s Milk Sensitivity in Adults
Source: Nutrients. 2021 Oct 27;13(11):3833. doi: 10.3390/nu13113833 (PMC8621065; doi:10.3390/nu13113833)

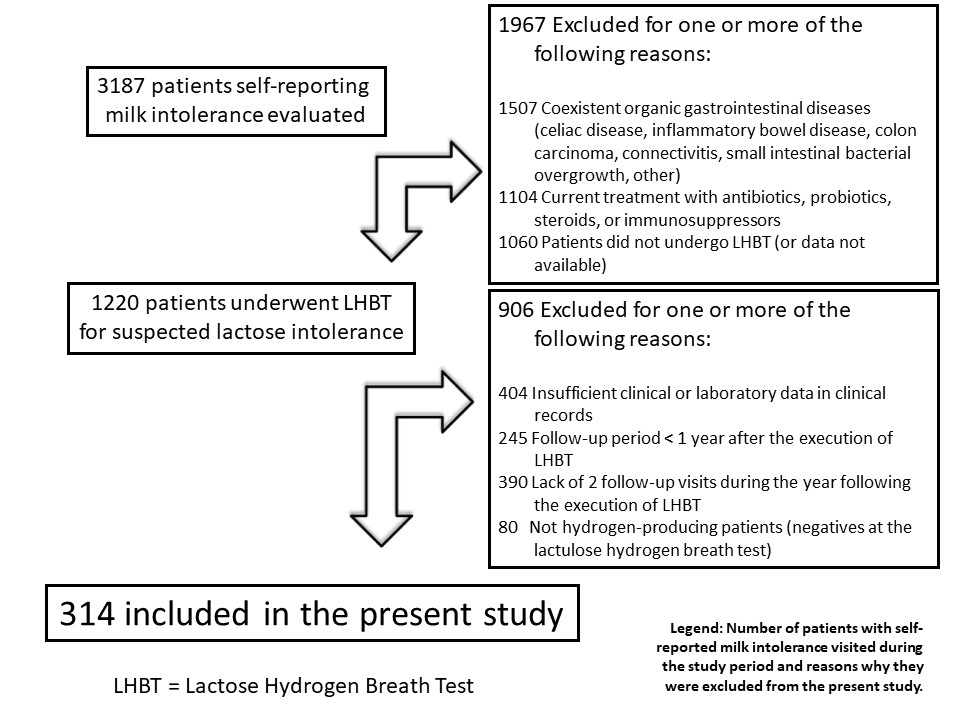

Supplement: Supplementary file 1 [file nutrients-13-03833-s001.zip › nutrients-1427360-supplementary/Carroccio et al - Supplementary file 2 October 1 2021.jpg]
